# Prevalence and Association of Human Papillomavirus with Esophageal Squamous Cell Carcinoma in Iran: A Systematic Review and Meta-Analysis

**Published:** 2019-07

**Authors:** Bashir MOHAMMADPOUR, Samaneh ROUHI, Mazaher KHODABANDEHLOO, Masoud MORADI

**Affiliations:** 1. Student Research Committee, Kurdistan University of Medical Sciences, Sanandaj, Iran; 2. Cellular & Molecular Research Center, Kurdistan University of Medical Sciences, Sanandaj, Iran; 3. Vice Chancellor for Research and Technology, Kurdistan University of Medical Sciences, Sanandaj, Iran

**Keywords:** Human papillomavirus (HPV), Esophageal cancer, Esophageal squamous cell carcinoma (ESCC)

## Abstract

**Background:**

Human papillomavirus (HPV) can infect the epithelium of the esophagus, but so far there is no reliable and comprehensive evidence about the prevalence and association of HPV with esophageal cancer in Iran, as high incidence region. This study aimed to evaluate the prevalence and association of HPV with esophageal squamous cell carcinoma (ESCC) in Iran.

**Methods:**

Relevant English and Persian articles published up to Aug 2017 and indexed in databases were reviewed. Frequency of HPV genotypes in ESCC cases and controls was surveyed according to regions of Iran. Data were meta-analyzed with random effects models using Comprehensive Meta-Analysis software.

**Results:**

Overall, 14 studies were eligible including 1444 samples (1062 ESCC cases and 382 controls). HPV was positive in 269 (25.32%) of 1062 ESCC cases and in 65 (17.01%) of 382 controls. Total prevalence of HPV in both groups was estimated 0.256 (95%CI, 0.208%–0.310%). The prevalence of HPV-16 and HPV-18 was estimated 0.121 (95%CI: 0.087–0.183) and 0.046 (95%CI; 0.023–0.088), respectively. The difference in HPV prevalence in different regions of Iran was statistically significant (Q=18.20, df =4, *P*=0.001). In 6 case-control studies, the pooled odds ratio was estimated 1.99 (95%CI; 0.916–4.315).

**Conclusion:**

High-risk HPVs were observed in ESCC cases and controls from different regions of Iran. The odds ratio indicates that the HPV infection in ESCC cases was approximately 2 fold more than the controls. More case-control studies in other populations with larger sample size are necessary.

## Introduction

Esophageal cancer (EC) is the most offensive malignancy of the gastrointestinal system and the eighth most common cancer worldwide (1). EC histologically has two subtypes, esophageal adenocarcinoma (EAC), and esophageal squamous cell carcinoma (ESCC). They vary in geographical distribution and etiology. ESCC is prevalent in developing countries (2,3). High incidence of EC has been seen in the region termed “Asian Esophageal Cancer Belt”, from Turkey, north provinces of Iran, Turkmenistan, China, and north of Russia (4,5).

The incidence of EC in different parts of Iran was surveyed by the Iran Cancer Institute, 9% of all malignancies and 27% of gastrointestinal malignancies were EC. Golestan Province is one of the high-risk areas, followed by Mazandaran and Khorasan Provinces, all in northeastern of Iran. The incidence of EC from 2005 to 2006 was 5.83 and 6.25 in males and females, respectively. Compatible with other areas of the world, ESCC constitutes more than 90% of all EC in northeastern of Iran (6,7).

EC is commonly diagnosed late when tumors have invaded peripheral tissues leading to dysphagia. Although, there are therapies for EC such as surgery and chemoradiation, prognosis of EC is weak. Nowadays, there are no screening methods to detect EC at early stages. Thus, recognition of etiology and applying preventive method is very important to control EC (2).

The probable cause of the EC consists of several risk factors that cooperate in a multi-stage process, including alcohol consuming, tobacco smoking, nutrition deficiencies, and infectious agents (1). In addition, some chemicals and lifestyle factors, including polycyclic aromatic hydrocarbons, opium use, hot tea drinking and genetic factors (oncogenes, tumor suppressor genes, cell cycle regulatory proteins) were associated with EC in north of Iran (2).

As a potential etiology of EC, that has been reported recently was human papillomavirus (HPV) infections, especially with high-risk genotypes including HPV-16 and HPV-18. The evidence of this relationship was weak and the results of these studies are not consistent (1). However, HPV infection is really associated with cervical, vaginal, vulvar, anal, penile, head and neck cancers (8).

The genome of HPV is a double-stranded circular DNA including early (E1 to E7) and late (L1, L2) genes or open reading frames (ORFs) encoding viral nonstructural regulatory proteins and viral structural proteins, respectively. Carcinogenesis of HPV is related to E6 and E7 which deregulate the cellular regulatory proteins (p53 and Rb), therefore leading to proliferation of the host cell. However, E2 has suppression effect on the E6 and E7 promoters. The genome of HPV integrates into the host cell chromosome in malignant tissues and disrupts E2 ORF. Therefore, decreased expression of E2 regulatory protein results in an increased expression of the viral E6 and E7 oncoproteins (9).

Using the sequence of L1 gene, about 200 HPV types have recognized. High risk HPV types including HPV-16 and 18 are more frequent in cancerous tissues, but other types [30, 31, 33, 35, 39, 45, 51–53, 56, 58, 59, 66, 68, 73, 82] are less associated with cancers (10).

Some meta-analyses were conducted to estimate the prevalence and association of HPV with ESCC worldwide (5, 11,12), but Persian language papers were not included in them.

In addition, molecular studies were conducted about the HPV effect on ESCC in some regions of Iran (4, 13–15). However, there is no reliable and comprehensive evidence about the prevalence and association of HPV with esophageal cancer in Iran, as high incidence region. We aimed to perform a systematic review and meta-analysis to determine the prevalence and association of HPV with esophageal cancer in Iran.

## Materials and Methods

### Literature search

All studies conducted on the prevalence of HPV in esophageal cancer in Iran were searched systematically from 2002 up to Aug 2017 for relevant articles published in English or Persian languages and indexed in databases such as PubMed, Google Scholar, Magiran, IranMedex, and Iranian Scientific Information Databank (SID). Keywords were “Human Papillomavirus”, “HPV”, “Esophageal Cancer”, “Esophageal Squamous Cell Carcinoma”, “ESCC” and “Iran”. Bibliographies and full texts of studies reporting HPV prevalence in esophageal cancers in Iran were entered into the EndNote software, according to the STROBE Statement-Checklist.

### Inclusion criteria

1. Studies reporting HPV infection in esophageal cancer and noncancerous tissues in Iran were selected; 2. only full-text studies in English or Persian articles were included.

### Exclusion criteria

1. Studies that the sampling location was unknown; 2. Studies that do not clearly outline the data; 3. Case report/series; 4. Letter to the editors; 5. Review articles; 6. Abstracts of conferences; 7. Studies that sampling took place at the same time and place reporting the same data.

### Selection and Data Extraction

Studies were identified based on the inclusion and the exclusion criteria from all English and Persian language articles reporting the frequency of HPV or its genotypes in esophageal cancers and controls in Iran. All appropriate articles were evaluated by two researchers, independently. With the exclusion of inappropriate studies, the data extracted and entered into an Excel file. If multiple publications reported the same data from the same population, only the publication with newer data was included. Extracted information were including first author, study location (province/city), publication year, if available dates of sample collection, sample size, gender, specimen type, frequency of esophageal cancers (cases), frequency of non-cancerous esophageal tissues (controls), HPV detection method, HPV genotyping method, overall and type-specific frequency of HPV in esophageal cancers (cases), or in non-cancerous esophageal tissues (controls), and grade of malignancy. For studies with samples from multiple province/city, data were extracted separately if possible.

### Statistical analysis

Data were analyzed using CMA (Comprehensive Meta-Analysis 2.2.064) software. The effect size was calculated as event rates and their 95% confidence interval. At first, the heterogeneity of the studies was evaluated using the Cochran’s Q and I^2^ indexes. The homogeneity was rejected; we used the random-effects model to analyze the publication bias using the *Egger* test and graph. To estimate the prevalence of HPV genotypes based on age, year of publication, and geographical area, meta-regression was used. At all steps, *P*-values below than 0.05 were considered statistically significant.

## Results

**Fig. 1:**
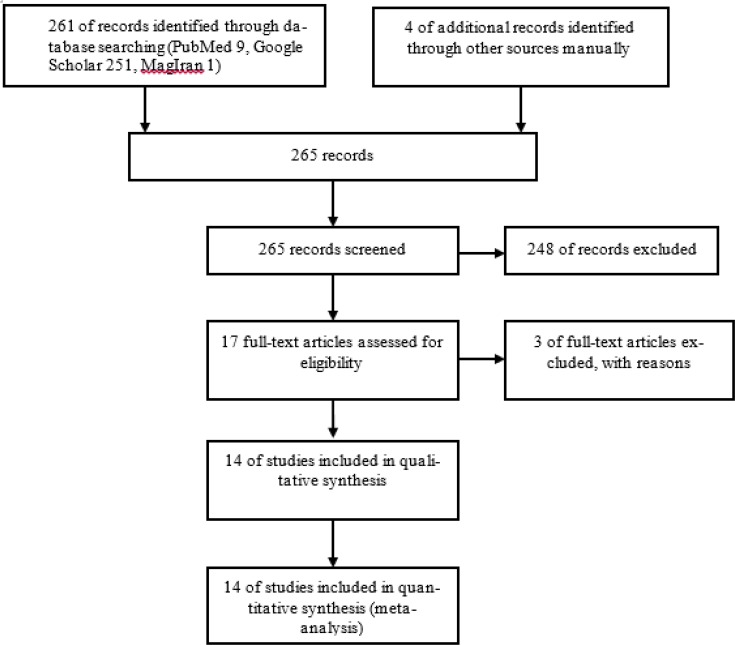
PRISMA diagram of study identification and selection

Based on the preliminary search strategies 261 studies were identified through database searching up to Aug 2017. In addition, four studies were found manually. From 265 studies screened, 248 studies were excluded, including unrelated articles, reviews, case reports, abstracts of conferences, and articles with incomplete information, based on the inclusion and exclusion criteria. Finally, 14 studies were eligible and were included in the meta-analysis (Fig. 1). Studies were categorized into five geographical regions of the country including Center (Tehran Province), North (Guilan, Mazandaran and Golestan provinces), South (Fars Province), East (Khorasan Razavi Province) and West (Kermanshah and Kurdistan provinces) (Table 1).

Total sample size in 14 studies was 1444, including 1062 esophageal squamous cell carcinomas (ESCC) cases, and 382 esophageal non-cancerous tissues (controls). Esophageal non-cancerous tissues had been taken from patients with dysphagia, endoscopically normal or margins of carcinomas. Specimen type of all of the samples had been biopsies from esophageal tissues, as formalin fixed and paraffin embedded (FFPE) tissue blocks.

In the studies that reported the age of patients, the age ranged from 22 to 91 yr in ESCC cases, and from 22 to 90 yr in the controls. In the studies that reported the gender of patients with ESCC; out of 1005 patients, 578 were men, and 427 were women; only one study (16) had not reported the gender of patients. Five studies reported the gender of non-cancerous patients, in which out of 192 patients, 95 were men and 97 were women, because of space restriction data were not shown in Table 1. The laboratory test used for DNA suitability in all of the studies was polymerase chain reaction (PCR) for the host cell beta-globin gene. In all studies, PCR had been conducted for detection of HPV using standard primers designed for viral L1 gene (GP5^+^/GP6^+^ and MY09/MY11), and E6 and E7 gene sequences. The laboratory methods used for detection of HPV genotypes were genotype specific PCR primers (E6/E7), sequencing of the PCR product, Real-time fluorescence detection (FRT) kit, restriction fragment length polymorphism (RFLP), and INNO-LiPA HPV genotyping kit (Table 1).

**Fig. 2:**
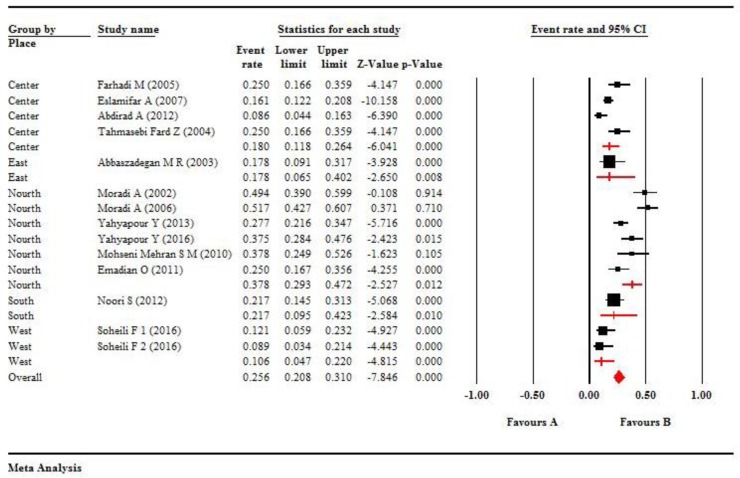
The forest plot shows meta-analysis on the overall prevalence of HPV infection in esophageal tissues according to regions of Iran. The squares represent pooled estimates of the prevalence of HPV infection, and lines show 95% confidence intervals. The diamond shows the overall prevalence of HPV infection in ESCC. The overall prevalence was estimated at 0.256 (95% CI, 0.208%–0.310%). *P*<0.001

Overall, 14 studies were eligible including 1444 samples (1062 ESCC cases and 382 controls). Out of 1444 samples, 334 (23.13%) patients were positive for HPV. In another word, HPV was positive in 269 (25.32%) of the 1062 ESCC cases and in 65 (17.01%) of 382 controls (Table 1). According to the values of I^2^=87.79, and *P*<0.001, we used random effect model in meta-analysis, thus the pooled overall prevalence of HPV was estimated 0.256 (95% CI, 0.208%–0.310%) in both groups (Fig. 2).

**Fig. 3:**
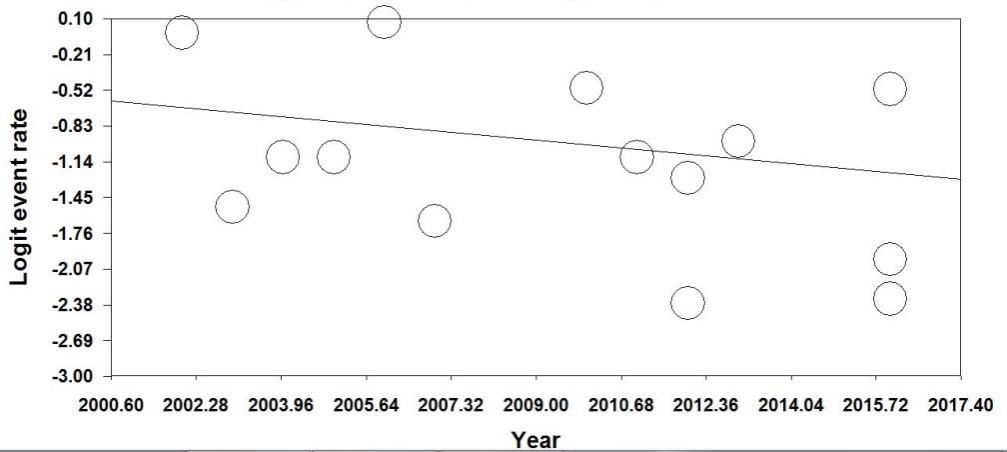
Regression of year on logit event rate of overall prevalence of HPV infection in patients with esophageal tissues, according to regression of publication years. According to the value of I^2^=80.25 and *P*<0.001. We used the random-effects model. Graph shows a decreasing trend

**Fig. 4:**
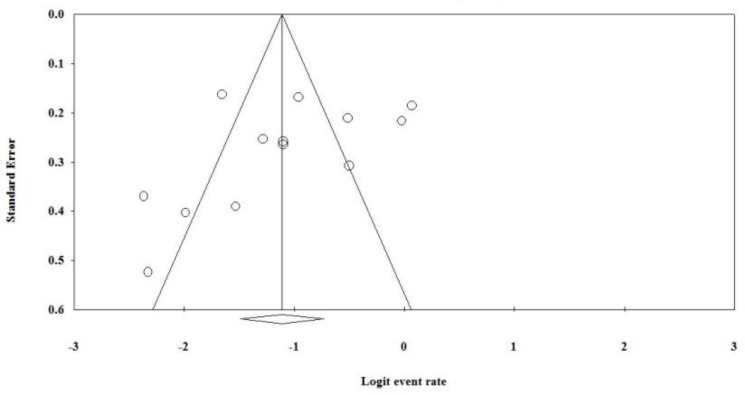
The funnel plot for analysis of publication bias for the overall prevalence of HPV infections in patients’ esophageal tissues, the figure shows publication bias

The difference in HPV prevalence in different regions of Iran was statistically significant (Q=18.20, df=4, *P*=0.001). According to regions of Iran, the maximum prevalence of HPV infection was 0.378 (95%CI, 0.293%–0.472%) in North of Iran (Guilan, Mazandaran, Golestan, Turkmen Sahra), as high incidence region (Fig. 2). Moreover, according to the value of I^2^ = 80.25 and *P*<0.001, we used the random effects model to estimate the overall prevalence of HPV, according to publication years. There was a decreasing trend in the overall prevalence of HPV, according to publication years (Fig. 3). However, there was an increasing trend in the overall prevalence of HPV according to patient’s ages. The funnel plot showed that there was publication bias (Fig. 4). Only five studies had reported the frequency of HPV infection, according to gender of patients with ESCC. Therefore, HPV was positive in 65 men and in 50 women with ESCC.

HPV genotypes had been reported separately or as combined infections in the most of the studies. Three studies had reported untypeable or unknown HPV genotypes. However, the genotypes of HPV in ESCC cases and controls according to each study are shown in Table 1. Most of them were high-risk HPV types. Total HPV genotypes are shown in the last row of Table 1.

**Table 1:** Studies reported the overall and genotypes of human papillomavirus (HPV) infection in patients with Esophageal Squamous Cell Carcinoma (cases) and Esophageal non-cancerous tissues (controls) in Iran

***Author (Year)***	***Province***	ESCC^*^ cases	Controls^**^	***HPV detection primers***	***Overall HPV in ESCCs***	***Overall HPV in Controls***	***Genotyping Method***	***HPV genotypes in ESCC cases (Frequency)***	***HPV genotypes in non-cancerous controls (Frequency)***	***Reference***
Farhadi M (2005)	Tehran	38	38	MY09/MY11	14	5	E6/E7 primers	HPV-16 (5), HPV-18 (3)	HPV-18 (5)	(17)
Eslamifar A (2007)	Tehran	140	140	GP5+/GP6+	33	12	Sequencing	HPV-16 (20), HPV-18 (10), HPV-33 (2), HPV-31 (1)	HPV-16 (12)	(18)
Abdirad A. (2012)	Tehran (Cancer Institute)	93	-	SPF10	8	-	INNO-LiPA genotyping kit	HPV-16 (2), HPV-6/16 (2), HPV-6/18 (1), HPV-6 (1), untypeable (3)	-	(19)
Haeri H. (2013)	Tehran (Cancer Institute)	30	30	Real-time GP5+/GP6+	-	-	-	-	-	(20)
Tahmasebi Fard Z. (2004)	Tehran	38	38	MY09/MY11	14	5	E6/E7 primers	HPV-16 (5), HPV-18 (3)	HPV-18 (5)	(21)
Abbaszadegan M.R (2003)	Khorasan Razavi	45	-	E6/E7 primers	8	-	E6/E7 primers	HPV-16 (8)	-	(22)
Moradi A. (2002)	Golestan (Turkmen Sahra)	85	-	GP5+/GP6+	42	-	Sequencing	HPV-16 (23), HPV-18 (2), HPV-52 (2), HPV-66 (3), HPV-6 (6), HPV-16/18 (2), HPV-16/6 (4)	-	(14)
Moradi A (2006).	Golestan (Turkmen Sahra)	85	31	GP5+/GP6+	42	18	Sequencing	HPV-16 (23), HPV-18 (2), HPV-52 (2), HPV-66 (3), HPV-6 (6), HPV-16/18 (2), HPV-18/6 (4)	HPV-16 (7), HPV-6 (8), HPV-66 (1), HPV-52 (2)	(15)
Yahyapour Y. (2013)	Mazandaran	177	-	Real-time MY09/MY11 kit	49	-	Real-time fluorescence detection (FRT) kit	HPV-6 (4), 18 (1), 11 (4), 39/45/59 (1), 16/45 (1), 39 (1), 11/31/33/35/56 (1), 39/45 (1), 6/39/45 (1), 11/45/52 (1), 35/52 (1), 31/33/52 (1), 52/58 (1), 39 (1), 52 (1), 45 (1), 56 (1), Unknown (26)	-	(23)
Yahyapour Y. (2016)	Mazandaran	51	45	Real-time MY09/MY11 kit	16	20	Real-time fluorescence detection (FRT) kit	HPV-11 (1), (HPV-16/45 (1), HPV-35/52 (1), HPV-39/45/59 (1), untypable (12)	untypable (11), HPV-11 (4), HPV-33 (1), 39 (1), 52 (1), 56 (1), 58 (1)	(4)
Mohseni S M. (2010)	Guilan	45	-	GP5+/GP6+	17	-	E6/E7 primers (Isogen)	HPV-16/18 (2), HPV-31/33/51/52/58 (4)	-	(24)
Emadian O. (2011)	Mazandaran	40	40	Nested PCR, Amplesence	15	5	E6/E7 primers, RFLP^****^, Amplesence	HPV-16 (6), HPV-16/45 (3), HPV-45 (6)	HPV-16 (3), HPV-45 (2)	(25)
Noori S. (2012)	Fars	92	20	GP5/GP6	-	-	-	-	-	(26)
Soheili F. (2016)	Kermanshah	58	-	GP5/GP6, SPF10	7	-	INNO-LiPA genotyping kit	HPV-16 (4), HPV-18 (1), HPV6/18 (2)	-	(9)
	Kurdistan	45	-	GP5/GP6, SPF10	4	-	INNO-LiPA genotyping kit	HPV-16 (2), HPV-18 (2)	-	
Total	-	1062	382	-	269	65	-	HPV-16 (117), HPV-18 (37), HPV-11 (7), HPV-31 (7), HPV-35 (2), HPV-39 (5), HPV-45 (11), HPV-52 (9), HPV-56 (2), HPV-58 (5), HPV-59 (2), HPV-66 (6), HPV-6 (20), untypable (41)	HPV-16 (22), HPV-18 (10), HPV11 (4), HPV-33 (1), 39 (1), HPV45 (2), HPV-52 (3), 56 (1), 58 (1), HPV-66 (1), HPV-6 (8), untypable (11)	-

* ESCC: Esophageal Squamous Cell Carcinoma,

** Biopsies from esophageal non-cancerous tissues from patients with dysphagia, endoscopically normal or margins of carcinomas as control;

*** All specimens were formalin fixed and paraffin embedded (FFPE) tissue blocks;

**** RFLP: Restriction Fragment Length Polymorphism

The maximum frequencies of HPV genotypes were HPV-16 and HPV-18 in the ESCC cases, 117 and 37, respectively. Moreover, HPV-16 and HPV-18 were higher in the controls, 22 and 10, respectively (Table 1).

**Fig. 5:**
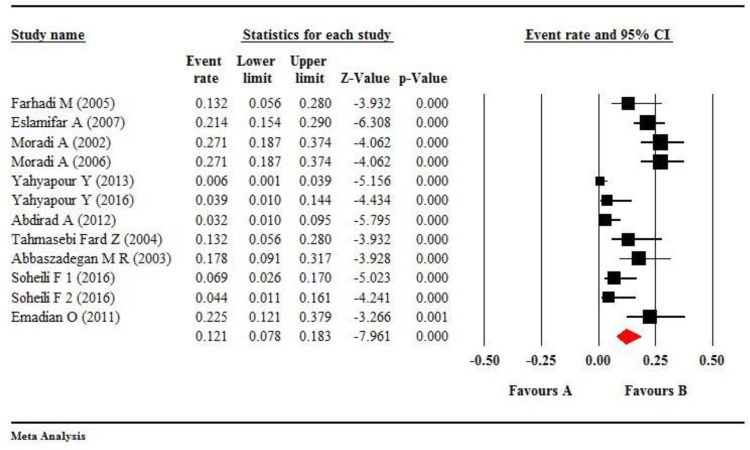
The forest plot shows meta-analysis on the prevalence of HPV-16 infection in esophageal tissues. The squares represent pooled estimates the prevalence of HPV-16 infection, and lines show 95% confidence intervals. The diamond shows the overall prevalence of HPV-16 infection. The overall prevalence was estimated at 0.121 (95% CI, 0.078%–0.183%)

The pooled prevalence of HPV-16 was estimated 0.121 (95%CI: 0.087–0.183) by using the random effect model (*P*<0.001, I^2^ =78.25) (Fig. 5). There was publication bias about HPV-16. The prevalence of HPV-16 was decreasing according to publication years (*P*<0.05) and with the age of patients (*P*<0.05). The difference in HPV-16 prevalence in different regions of Iran was not statistically significant (Q=1.84, df=3, *P*=0.61).

**Fig. 6:**
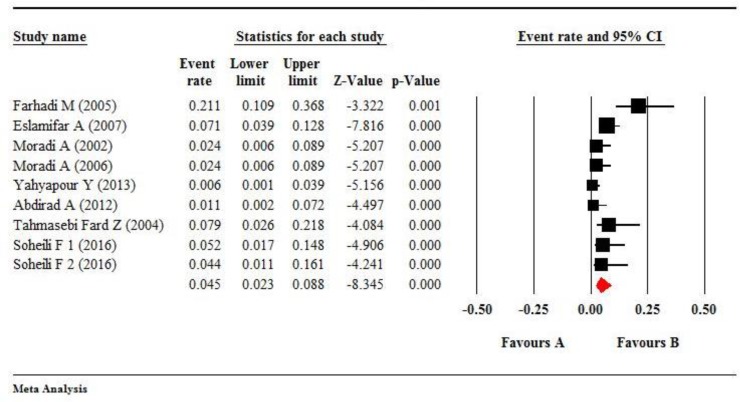
The forest plot shows meta-analysis on the prevalence of HPV-18 infection in esophageal tissues. The squares represent pooled estimates the prevalence of HPV-18 infection and lines shows 95% confidence intervals. The diamond shows the overall prevalence of HPV-18 infection that was estimated at 0.046 (95% CI, 0.27%–0.076%)

The pooled prevalence of HPV-18 was estimated at 0.045 (95%CI; 0.023–0.088) by using the random effect model (*P*<0.001, I^2^=58.368) (Fig. 6). In addition, there was publication bias about HPV-18. The prevalence of HPV-18 was decreasing according to publication years and with the ages of patients. The difference of HPV-18 prevalence in different regions of Iran was not statistically significant (Q=5.43, df=2, *P*=0.07).

**Fig. 7:**
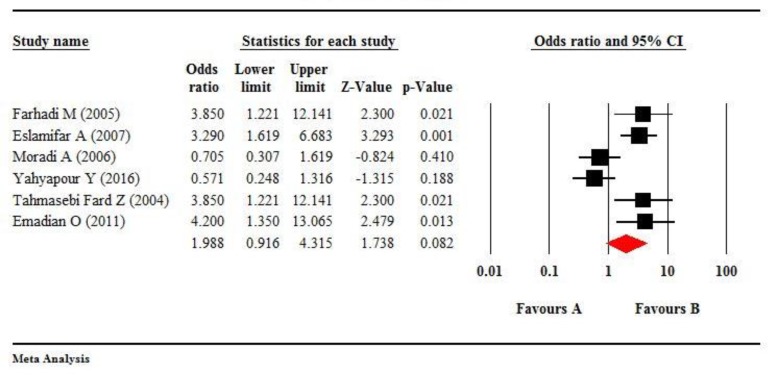
The forest plot of meta-analysis on overall HPV infection odds ratio and esophageal squamous cell carcinoma (ESCC) risk in six eligible case-control studies. The squares show pooled estimates of the HPV infection odds ratio and ESCC risk, and the lines show 95% confidence intervals. The diamond shows the pooled odds ratio that was estimated at 1.988 (95% CI: 0.716–4.438)

Non-cancerous controls had been included in 8 of the reviewed studies; but, the frequency of HPV in ESCC cases was zero in two studies (Table 1). Therefore, only 6 studies were analyzed as case-control. According to the values of I^2^=75.55 and *P* =0.001, we used the random-effects model. The estimated pooled odds ratio (OR) was 1.988 (95%CI; 0.916, 4.315), which indicates that the exposure ratio in ESCC cases was approximately two-fold more than the controls. However, this ratio was not statistically significant with respect to the confidence interval (*P*=0.08) (Fig. 7).

Overall, 8 studies had reported the histopathological degree of ESCC in the patients, including 141 well differentiated, 182 moderately differentiated, 91 poorly differentiated, and 33 not differentiated. The histopathological degree of ESCC cases was not statistically significant with respect to study regions, years of publication and ages of patients.

## Discussion

We investigated the prevalence and relation of HPV infection with esophageal squamous cell carcinoma (ESCC) in Iran. Overall, 14 studies were eligible including 1444 samples (1062 ESCC cases and 382 controls). Out of 1444 samples, 334 (23.13%) patients were positive for HPV. The total prevalence of HPV in both groups was estimated 0.256 (95% CI, 0.208%–0.310%). HPV-16 was the most frequent type with a pooled prevalence of 0.121 (65%CI: 0.087–0.183). The frequency of HPV in ESCC cases and non-cancerous controls were 318 (25.32%) and 65 (17.01%), respectively. In six case-control studies, the risk or odds ratio (OR) was estimated at 1.988 (95% CI: 0.716–4.438). The maximum prevalence of HPV infection was in North of Iran, with a higher distribution of esophageal cancer (EC). This study provides comprehensive evidence of HPV prevalence and association of HPV with ESCC in Iran.

In a meta-analysis including 17 studies, the prevalence of HPV in Iranian patients with gastrointestinal cancers was 16.4% (95% CI: 10.4–24.9). Considering all HPV genotypes, the OR of gastrointestinal cancers in positive cases was 3.03 (95%CI: 1.42–6.45), but in HPV-16 positive patients OR was 3.62 (95% CI: 1.43–4.82) (13). The reason for differences in results may be due to that, we focused only on EC.

A systematic review and meta-analysis was conducted on the association of HPV-16 and HPV-18 with EC worldwide. In 33 randomized studies HPV infection rate in the EC group was 46.5%, while HPV infection in the control group was 26.2%, (OR: 1.62, 95% CI: 1.33–1.98). There were statistical differences in HPV detection methods, including PCR, immunohistochemistry (IHC) and in situ hybridization (ISH). The merged OR was 1.61 (95% CI: 1.33–1.95) in the PCR detection group. The authors concluded that HPV infection closely associated with EC (1).

In a meta-analysis and systematic review, the association of HPV-16 infection and EC in Chinese population was studied. Overall, 1442 EC cases and 1602 controls from 10 eligible studies, the estimated pooled OR was 6.36 (95% CI: 4.46, 9.07). In sensitivity analysis, the estimates for OR ranged from 5.92 (95% CI: 4.08, 8.60) to 6.97 (95% CI: 4.89, 9.93). HPV-16 infection may be a risk factor for the EC among Chinese population (27).

Overall, 3429 EC cases were evaluated from 26 eligible studies in China. The pooled estimate of HPV-16 prevalence was 0.381 (95% CI: 0.283, 0.479). The prevalence varied by geographical regions, publication year, HPV detection method and specimen type. A relatively high level of HPV-16 prevalence was in the EC among Chinese population (12).

A meta-analysis was conducted worldwide, including 124 studies with 13 832 ESCC cases. The estimated prevalence of HPV in ESCC cases was 0.277 (95% CI: 0.234, 0.320) by PCR; 0.243 (0.159, 0.326) by ISH; 0.304 (0.185, 0.423) by IHC; 0.322 (0.154, 0.490) by L1 serology; and 0.176 (0.061, 0.292) by Southern blot assay. The highest HPV prevalence was occurred in Africa and Asia, especially in China. Case-control studies should be done on the quantifying of HPV in ESCC cases and detection the association between HPV and the incidence of ESCC in large populations for a dipper understanding of the etiologic role of HPV on ESCC (5).

In a meta-analysis 76 studies, including 8990 patients with ESCC and 174 patients with esophageal adenocarcinomas (EA), pooled HPV prevalence in ESCC cases was 22.2% (95% CI, 18.3%–26.7%). HPV-16 was the most frequent type with a pooled prevalence of 11.4% (95% CI: 8.2%–15.7%). HPV prevalence in EA cases was 35.0% (95% CI, 13.2%–65.7%) and prevalence of HPV-16 was 11.4% (95% CI: 8.2%–15.7%). The association between HPV infection and ESCC was significant with a pooled OR of 3.32 (95% CI, 2.26–4.87). With respect to HPV-16, the OR of the association was 3.52 (95% CI, 2.04–6.07). HPV infection was associated with an increased risk of ESCC. However, due to the heterogeneity of studies and weaker association in comparison to cervical and laryngeal cancer, more studies are needed to clarify the relation of HPV and ESCC (28).

A meta-analysis was performed on 132 studies about the relation of HPV and ESCC. An increased risk of ESCC in patients with HPV was found (OR: 2.69, 95% CI 2.05–3.54). The prevalence of HPV in ESCC cases was 24.8%. The risk of ESCC associated with HPV-16 infection was high (OR: 2.35, 95% CI 1.73–3.19). This risk of association was higher in Asia (OR 2.94, 95% CI 2.16–4.00), particularly China (OR 2.85, 95% CI 2.05–3.96). The authors concluded that an increased infection of HPV occurred in ESCC cases (29).

In a meta-analysis including 5755 ESCC cases from 68 studies, 11.67% (95% CI, 7.74%–16.21%) of ESCC cases had HPV-16 and 1.82% (95% CI, 0.90%–2.95%) had HPV-18. In 10 case-control studies a significant increase in ESCC risk was observed for HPV-16, summary OR was 3.55 (95% CI, 2.05%–6.14%), but no significant increase for HPV-18, summary OR was 1.25 (95% CIs, 0.46%–3.43%). HPV-16 and HPV-18 can be detected in ESCC cases and HPV-16, but not HPV-18, was associated with the risk of ESCC (30).

In a meta-analysis, 152 eligible studies were included and 10 234 ESCC cases were analyzed for HPV infection by different detection techniques in different geographical regions. Out of 10 234 cases, 3135 (30.6%) were positive for HPV, with an effect size of 0.29 (95% CI 0.251–0.31) using random effects model. A significant heterogeneity observed between the studies (*P*=0.440) from the different regions. Wide variability in HPV rates in ESCC cases is not due to the detection methods but is due to the geographic region of the study. ESCC might have different etiologies in low-incidence and high-incidence regions, and HPV was an important role in the latter regions (11).

A meta-analysis included 21 case-control studies (1223 ESCC cases and 1415 controls). From all ESCC cases, 426 (35%) were positive for HPV. The pooled OR for the association of HPV and ESCC was estimated at 3.04 (95% CI, 2.20 to 4.20). The effect of individual studies on the pooled estimation was non-significant. In countries with lower to medium ESCC incidence the relationship was stronger (OR 4.65, 95% CI 2.47 to 8.76) than regions with high ESCC incidence (OR 2.65, 95% CI 1.80 to 3.91). It was concluded that hesitancy about the role of HPV in ESCC was due to the low number of well-designed studies. In addition, HPV infection increases the risk of ESCC 3-fold and vaccination could prevent ESCC in high incidence countries (3).

A study determined the associations between HPV and ESCC by measuring serological markers of HPV in the sera of patients. Serum samples were tested in 1561 ESCC cases and 2502 controls from 6 case-control studies for antibodies to the HPV major capsid protein (L1) and/or the HPV early proteins (E6 and/or E7). Statistically significant associations were observed between ESCC and antibodies to E6 of HPV-16 (OR 1.89, 95% CI 1.09 to 3.29, *P*=0.023) and HPV-6 (OR 2.53, 95% CI 1.51 to 4.25, *P*<0.001). No significant associations were observed between ESCC and antibodies to E7 of the HPV types. In addition, significant associations was between ESCC and antibodies to L1 capsid protein of HPV-33 (OR 1.30, 95% CI 1.00 to 1.69; *P*=0.047), HPV-6 (OR 1.22, 95% CI 1.05 to 1.42, *P*=0.010) and HPV-11 (OR=1.30, 95% CI=1.09 to 1.56, *P*=0.0036). There was limited serological evidence of an association between ESCC and HPV in their population (31).

A study reported the prevalence of HPV in oral of healthy individuals and relative risk factors in Iran. Saliva samples of 114 healthy individuals were collected for HPV DNA detection. The frequency of oral HPV was 7 (6.1%) in healthy individuals. Virus type in five of them was HPV-18. Moreover, HPV-6 and HPV-66 types were detected in two cases. Relation of oral HPV positivity to demographic data and risk factors was not statistically significant (32).

Power of association between HPV and esophageal cancer has not been as strong as detected in cervical and laryngeal cancer (28). This may be due to the small number of appropriately designed studies (3), such as case-control studies with large sample sizes (5).

In comparison, the results of HPV prevalence and its association with ESCC in the present study were somewhat similar to some previous studies. The differences of results of in the present study may be due to geographical region, environmental factors, differences of the population (race, living habits), research design, sample size, type of specimen, statistical analysis, and heterogeneity of included studies.

## Conclusion

High-risk human papillomaviruses were observed in ESCC tissues and controls from different regions of Iran. The odds ratio indicates that the HPV in ESCC cases was approximately two-fold more than the controls. With respect to the defined oncogenic role of HPV in some cancers such as cervical cancers, the causality of this virus in ESCC needs more case-control studies in other populations with larger sample size.

## Ethical considerations

Ethical issues (Including plagiarism, informed consent, misconduct, data fabrication and/or falsification, double publication and/or submission, redundancy, etc.) have been completely observed by the authors.
